# Procalcific Phenotypic Drift of Circulating Progenitor Cells in Type 2 Diabetes with Coronary Artery Disease

**DOI:** 10.1155/2012/921685

**Published:** 2012-02-28

**Authors:** Gian Paolo Fadini, Mattia Albiero, Lisa Menegazzo, Elisa Boscaro, Carlo Agostini, Saula Vigili de Kreutzenberg, Marcello Rattazzi, Angelo Avogaro

**Affiliations:** ^1^Department of Clinical and Experimental Medicine, University of Padova, 35128 Padova, Italy; ^2^Venetian Institute of Molecular Medicine, Laboratory of Experimental Diabetology, 35129 Padova, Italy; ^3^Dipartimento di Medicina Clinica e Sperimentale, Policlinico Universitario VIII piano, Via Giustiniani, 2, 35100 Padova, Italy

## Abstract

Diabetes mellitus (DM) alters circulating progenitor cells relevant for the pathophysiology of coronary artery disease (CAD). While endothelial progenitor cells (EPCs) are reduced, there is no data on procalcific polarization of circulating progenitors, which may contribute to vascular calcification in these patients. In a cohort of 107 subjects with and without DM and CAD, we analyzed the pro-calcific versus endothelial differentiation status of circulating CD34+ progenitor cells. Endothelial commitment was determined by expression of VEGFR-2 (KDR) and pro-calcific polarization by expression of osteocalcin (OC) and bone alkaline phosphatase (BAP). We found that DM patients had significantly higher expression of OC and BAP on circulating CD34+ cells than control subjects, especially in the presence of CAD. In patients with DM and CAD, the ratio of OC/KDR, BAP/KDR, and OC+BAP/KDR was about 3-fold increased than in other groups. EPCs cultured from DM patients with CAD occasionally formed structures highly suggestive of calcified nodules, and the expression of osteogenic markers by EPCs from control subjects was significantly increased in response to the toll-like receptor agonist LPS. In conclusion, circulating progenitor cells of diabetic patients show a phenotypic drift toward a pro-calcific phenotype that may be driven by inflammatory signals.

## 1. Introduction

Diabetes mellitus (DM) is associated with an excess risk of cardiovascular disease, which is attributable to hyperglycemia, oxidative stress, and inflammation [1]. In parallel, repair of vascular damage is compromised in DM owing to a pauperization of circulating endothelial progenitor cells (EPCs) [2]. In the bloodstream, other lineage-committed progenitor cells may have pathophysiological implications in the cardiovascular system, such as smooth muscle and cardiomyocyte progenitors [3, 4]. DM reduces circulating EPC level [5] and is also associated with an impaired generation of EPCs paralleled by enhanced differentiation of smooth muscle progenitors in vitro [6, 7]. A drift of circulating progenitors from the endothelial to smooth muscle-cell phenotype has been linked to the development of myointimal hyperplasia [8], an event that is associated with diabetes. The impaired differentiation of cultured EPCs in diabetic patients has been previously attributed to a proinflammatory status [9]. EPCs promote endothelial healing and compensatory angiogenesis, thus providing a mean of vascular repair [10]. Therefore, abnormalities of progenitor cells are considered important contributors to the development of diabetic vasculopathy, which is characterized by extensive endothelial dysfunction/damage and myointimal hyperplasia [11]. Another hallmark feature of diabetic vasculopathy is ectopic calcification. Intimal microcalcifications of atherosclerotic lesions contribute to destabilize the plaque, while medial calcification rises arterial stiffness and blood pressure [12, 13]. The mechanisms increasing vascular calcification in diabetes are incompletely understood, but cell-mediated processes are increasingly studied. In 2005 Eghbali-Fatourechi and coworkers described the existence of osteoblastic cells in the human peripheral blood [14], suggesting for the first time that circulating cells may contribute to ectopic calcification. This hypothesis has been supported by the discovery of myeloid calcifying cells (MCCs) and by evidence of a potential role for circulating cells in vascular and valve calcification [15–17].

Based on this background, we hypothesize that circulating progenitor cells of diabetic patients may undergo a phenotypic shift from the protective endothelial commitment to a detrimental pro-calcific phenotype. To test this hypothesis, we analyzed expression of endothelial and osteogenic markers on circulating CD34+ cells and evaluated pro-calcific differentiation of EPCs in vitro.

## 2. Materials and Methods

### 2.1. Patients

The study was approved by the Ethical Committee for Clinical Experimentation of the University Hospital of Padova. Type 2 diabetic patients and controls were recruited at the outpatient clinics of the Division of Metabolic Diseases. The same exclusion criteria applied to all patients: age <18 or >80 years; recent (within 1 month) trauma, surgery, or revascularization; immunological disease, immunosuppression, or cancer; any acute disease or infection; pregnancy and lactation. Patients were divided according to the presence of diabetes mellitus (DM) and coronary artery disease (CAD). Type 2 DM was diagnosed according to American Diabetes Association criteria [18]. CAD was defined in the presence of at least one of the followings: a past documented history of myocardial infarction; angiographic evidence of one or more >70% stenosis of epicardial coronary arteries; evidence of inducible ischemia from a noninvasive stress test (either single-photon emission tomography or ultrasound Doppler examination). All patients were characterized by collection of the following data: age, sex body mass index (kg/m^2^), systolic and diastolic blood pressure, diagnosis of hypertension, history of smoking, fasting plasma glucose, glycated hemoglobin (HbA1c), total cholesterol, HDL cholesterol, and triglycerides concentration. We also recorded data on diabetic complications, included retinopathy (defined by the ETDRS classification [19]), neuropathy (defined by suggestive symptoms and signs, eventually confirmed by an electromyogram), and nephropathy (defined as either a urinary albumin excretion rate >30 mg/g creatinine or an estimated glomerular filtration rate [eGFR, according to the MDRD equation [20]] <60 mL/min/m^2^). Peripheral arterial disease was defined as a history of claudication or rest pain in the presence of a significant stenosis of leg arteries on an ultrasound or angiographic examination. Cerebrovascular disease was defined as either a history of past stroke/transient ischemic attack, or evidence of carotid atherosclerotic plaques, determining a stenosis >20% of vessel lumen, on an ultrasound examination. Finally, we also collected data on medications.

### 2.2. Cell Culture

Late outgrown EPCs were cultured from peripheral blood mononuclear cells as previously described [6]. Briefly, cells were plated on six-well fibronectin-coated plates at a density of 6 × 10^6^ cells per well and grown in supplemented endothelial cell growth medium (Clonetics) with 20% serum. The medium was changed the first time after 4 days and then each other day for a total of 2 weeks. We have previously shown that during the culture protocol these cells form clusters with a core made of rounded cells and radiating spindle-shaped cells at the periphery. At 12–14 days, these clusters dissolve and cells progressively develop as a monolayer. We characterized these cells by double immunofluorescence; cells were incubated at 37°C with 1 mg/mL Di-I-AcLDL (DiIacetylated low-density lipoproteins, Molecular Probes) for 1 h, followed by dark incubation with 15 mg/mL FITC-conjugated Ulex lectin (Sigma-Aldrich) for 2 h. Nuclei were stained in blue with Hoechst 33258 (Sigma-Aldrich). In separated experiments, cells were cultured in the presence of LPS (Sigma-Aldrich) from day 7 to 14 at a final concentration of 100 nM; untreated cells served as controls, and expression level was set at 1. In parallel we also cultured human umbilical vein endothelial cells (HUVECs, Clonetics) and analyzed the expression of bone-related markers in untreated and LPS-treated cells. Experiments were performed in triplicate. Alizarin red and von Kossa stainings were not performed.

### 2.3. Flow Cytometry

Expression of progenitor cell antigens and differentiation markers was analyzed by multicolor flow cytometry on fresh whole peripheral blood samples. Briefly, after red blood cell lysis, cells were incubated with specific monoclonal antibodies anti-CD34 (-PE or -FITC conjugated, Becton Dickinson, BD), PE-conjugated anti-KDR (R&D Systems), or PE-conjugated anti-OC (R&D Systems) and APC-conjugated anti-BAP (R&D Systems). OC/BAP costaining with KDR was not performed. After washing, cells were analyzed by FACSCalibur instrumentation (BD) set up for analysis or rare events. We first gated CD34+ cells in the mononuclear cell fraction and then examined the resulting population for dual expression of KDR or dual/triple expression of OC and/or BAP. At least 5 × 10^5^ events were acquired, and positive events were recorded as a fraction of the number of fated CD34+ cells. All analyses were performed by trained operators blinded to the patients status. For the analysis of cell culture, a similar gating strategy was used, with the same directly labelled monoclonal antibodies plus the PE-conjugated anti-RANKL mAb.

### 2.4. Statistical Analysis

Data are expressed as mean and standard error or as percentage, where appropriate. Comparison between 2 or more groups was performed using Student's *t* test or ANOVA, respectively. The Least Significance Difference (LSD) post hoc test was used. Comparison of categorical data was tested using the Chi square test. To test the independent association of the coexistence of DM and CAD on progenitor cell phenotypes, we run a multiple linear regression analysis in which DM+CAD+ was an independent variable together with other covariates. Covariates were selected for being different at *P* < 0.05 at the univariate comparison between patients with and without DM and CAD. Statistical significance was accepted at *P* < 0.05, and the SPSS versus 16.0 was used.

## 3. Results

### 3.1. Patients' Characteristics

A total of 107 subjects were included in the study. They were divided into 4 groups according to the presence of DM and/or CAD. Sample size was fairly balanced among groups. Among patients without DM, patients with CAD had a higher prevalence of the male gender, lower HDL cholesterol, and a much larger use of cardiovascular medications than in those without. Obvious differences were detected in patients with DM than in those without, including older age, prevalence of males, higher BMI, blood pressure, cholesterol, plasma glucose and HbA1c, comorbidities, and medications. Interestingly, among DM patients, the presence of CAD was only associated to a significantly lower HDL cholesterol and higher prevalence of nephropathy (Table 1).

### 3.2. Expression of Bone-Related Markers on Circulating Progenitor Cells

**Figure 1:**
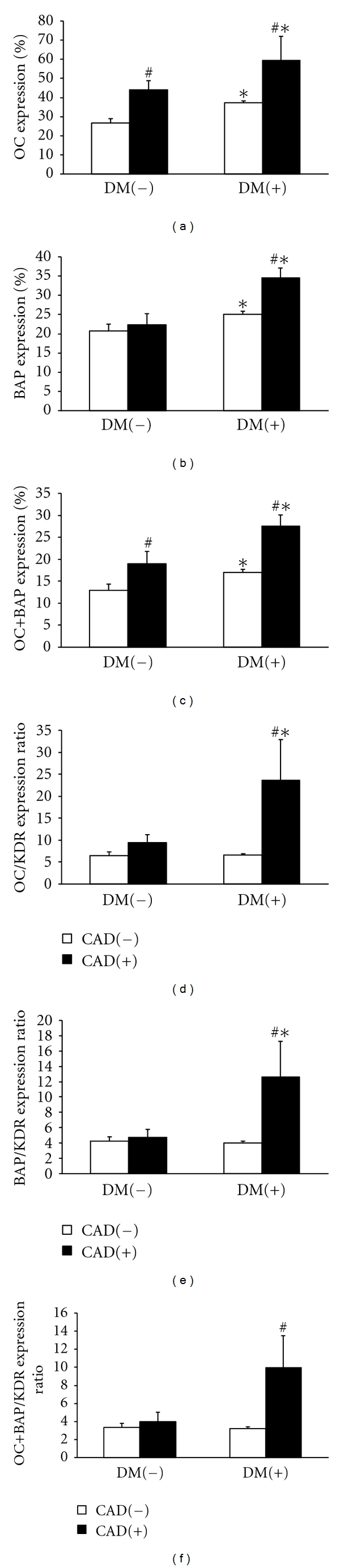
Expression of bone-related markers on circulating CD34+ progenitor cells. Patients were divided according to the presence/absence of type 2 diabetes mellitus (DM) and coronary artery disease (CAD). Post hoc tests: *P < 0.05 in DM+ versus DM−; ^#^ *P* < 0.05 in CAD+ versus CAD−.

To detect the pro-calcific differentiation of circulating CD34+ progenitor cells, we analyzed the expression of OC and BAP. In CD34+ cells from control healthy subjects (DM−CAD−), OC was expressed on 26.7 ± 2.1% while BAP was expressed on 20.8 ± 1.7% of cells, and the coexpression of both markers was 12.9 ± 1.4%. The expression of OC and/or BAP was significantly increased in patients with DM and/or CAD. Specifically, OC expression was higher in CAD versus non-CAD patients independently of DM, and in DM versus non-DM patients independently of CAD. BAP expression was higher in DM versus non-DM patients, especially in the presence of CAD. Co-expression of OC and BAP on CD34+ cells was significantly higher in both DM and CAD patients (Figures 1(a), 1(b), and 1(c)).

### 3.3. Procalcific Phenotypic Drift of Circulating Progenitors

In parallel to the analysis of bone-related markers, we also examined the extent to which circulating CD34+ progenitor cells express the endothelial antigen KDR, which functionally represents type 2 VEGF receptor and is usually taken to represent endothelial differentiation [21]. This was used to determine the ratio of bone versus endothelial marker expression on CD34+ cells, as an indicator of a phenotypic drift of circulating progenitors toward the pro-calcific phenotype. We found that OC/KDR, BAP/KDR, and OC+BAP/KDR expression ratio was increased in DM+CAD+ patients versus controls by 3.6-, 2.9-, and 3.0-fold, respectively, while there were no differences among other groups (Figures 1(d), 1(c), 1(e), and 1(f)). Upon a multiple regression analysis, the coexistence of DM and CAD remained significantly associated with increased OC/KDR, BAP/KDR, and OC+BAP/KDR expression ratio versus other patients, independently of age, prevalence of hypertension, concentrations of total cholesterol, HDL and LDL, which were significantly different between the two groups (Delta ± SE 16.7 ± 6.1 for OC/KDR, *P* = 0.08; 8.7 ± 4.0 for BAP/KDR, *P* = 0.030; 6.8 ± 2.9 for OC+BAP/KDR, *P* = 0.023).

### 3.4. Calcification and Expression of Bone-Related Markers in Cultured EPCs

**Figure 2:**
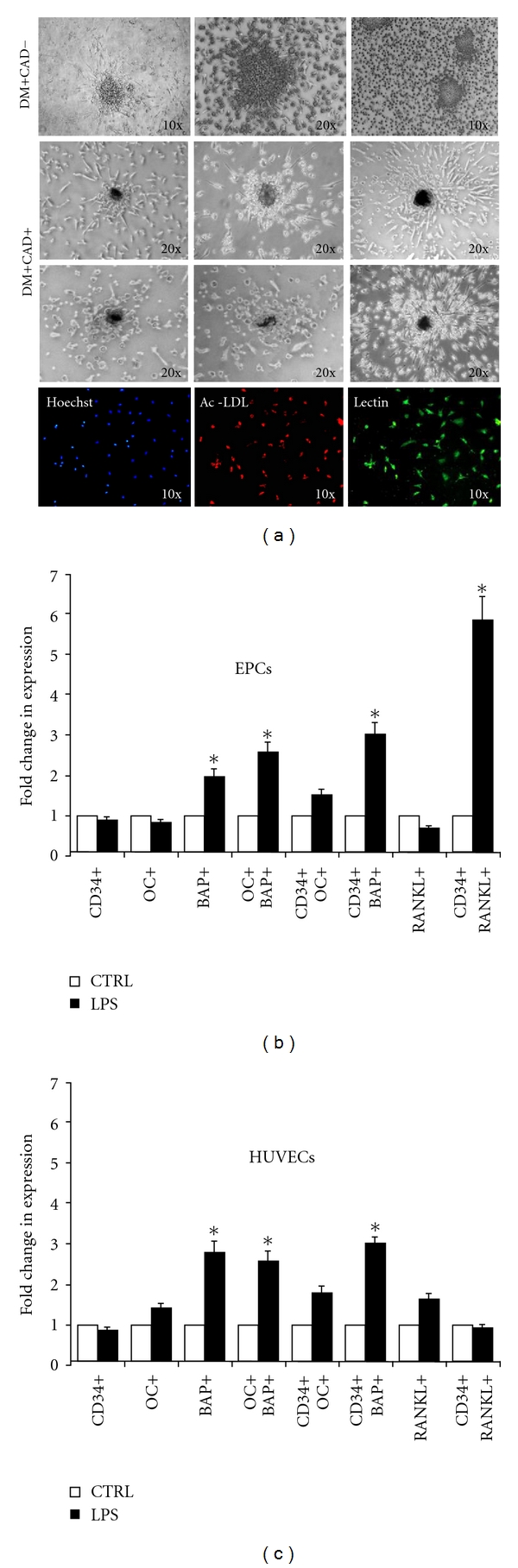
Pro-calcific differentiation of cultured EPCs. (a) EPCs cultured from DM+CAD+ patients developed dense nodules highly suggestive of calcification, while EPCs cultured from DM+CAD− patients did not. The lower lane shows double Lectin/AcLDL immunofluorescence analysis of cultured EPCs. ((b) and (c)) Cultured EPCs and HUVECs express bone-related antigenic markers after challenging with LPS 100 nM for 7 days. *P < 0.05 versus control experiment, set at 1.0.

To assess whether endothelial progenitors cultured from peripheral blood mononuclear cells can undergo a pro-calcific differentiation, we isolated late EPCs from diabetic patients. Extensive characterization of these cells is reported elsewhere [6, 22]. Clusters of EPCs occasionally formed dense nodules that were highly suggestive of calcification only when cultured from DM+CAD+ patients and not from DM+CAD− patients (2/6 versus 0/7, *P* = 0.05, Figure 2(a)). As EPCs express the LPS receptors CD14 and toll-like receptor-2 (TLR-2) [23], we tested whether challenging EPCs isolated from DM-CAD subjects with LPS resulted in upregulation of bone-related markers. We found that LPS significantly increased 2.6-fold OC+BAP+ cells in the culture and upregulated BAP (3.0-fold) and RANKL (5.8-fold) on CD34+ cells. In HUVECs, which served as a control cell type, there were similar increases in OC+BAP+ cells (2.6-fold), and expression of BAP (2.9-fold), but there was no change in expression of the osteoblast marker RANKL (Figure 2(b)).

## 4. Discussion

In the present study, we demonstrate for the first time that circulating progenitor cells from diabetic patients with coronary artery disease undergo a pro-calcific phenotypic shift, as evidenced by increased expression of bone-related markers versus endothelial markers.

In recent years, evidence accumulated in support of the existence of circulating progenitors for several lineages important for the cardiovascular system, including endothelial (EPCs), smooth muscle, and cardiomyocyte progenitor cells [3, 4, 24]. EPCs are by far the most extensively characterized of these circulating progenitors; they are defined by co-expression of immaturity (e.g., CD34) and endothelial (e.g., KDR) antigens [25]. About 10–15% of circulating CD34+ express KDR, the %KDR expression is usually taken to represent the extent to which circulating progenitors are committed to the endothelial lineage [21]. Recent data have demonstrated that circulating CD34+ progenitor cells and CD34+KDR+ EPCs can also express bone-related proteins, especially OC [26, 27]. Several preclinical studies and preliminary clinical evidence indicate that EPCs home to sites of vascular damage [28, 29]. Therefore, an osteogenic differentiation of these cells may be involved in the process of vascular calcification. Gössl et al. have found that OC expression on circulating EPCs is significantly associated with CAD in a cohort of patients with a very low prevalence of diabetes (7/72, 10%) [27].

Subsequently, they have demonstrated that OC-expressing EPCs are retained in the coronary circulation of patients with coronary endothelial dysfunction, providing an indirect evidence in support of homing of these pro-calcific cells at sites of vascular damage [26]. We have previously shown that OC+BAP+ myeloid calcifying cells (MCCs) are increased in the bloodstream and in calcified atherosclerotic lesions of type 2 diabetic patients [15], providing the first evidence that circulating cells may contribute to ectopic vascular calcification. However, so far, there was no data on pro-calcific differentiation of circulating progenitor cells in diabetic patients. This is of paramount importance because diabetes is typically associated with an exceedingly high prevalence of vascular calcification, either medial or intimal [30]. Herein, we show that expression of OC and BAP on CD34+ cells is increased in patients with either DM or CAD and that the coexistence of DM and CAD is associated with an almost doubled expression of these bone-related proteins. OC is a noncollagenous bone protein implicated in bone mineralization and calcium homeostasis, while BAP is a tetrameric glycoprotein found on the surface of osteoblast cells, and its function is essential to the mineralization process. If these cells are recruited to sites of vascular damage, it is easy to anticipate how they may promote the process of vascular calcification. Importantly, we have previously shown that the expression of KDR on CD34+ cells is reduced in diabetic patients with macroangiopathy, indicative of an impaired endothelial differentiation and generation of EPCs. Together with the enhanced osteogenic polarization, data consistently suggest that circulating progenitor cells of diabetic patients undergo a phenotypic drift toward the detrimental osteogenic phenotype at the expenses of the vasculoprotective endothelial phenotype. To quantitatively support this hypothesis, we examined the expression ratio of bone-related markers OC and BAP over KDR on circulating CD34+ cells. We found that OC/KDR, BAP/KDR, and OC+BAP/KDR are markedly elevated only in patients with DM and CAD and not in patients with either conditions, strengthening the association between this pro-calcific drift and diabetic vascular disease.

The degree of pro-calcific differentiation of CD34+ cells resembles the extent of OC and BAP expression on circulating monocytes and the levels of MCCs [15], suggesting that the driving force of the osteogenic program acts similarly on different cellular populations. To study the pro-calcific polarization of progenitor cells in vitro, we cultured late outgrown EPCs and found that they formed hyperdense nodular structures, highly suggestive of calcifications, only in DM+CAD+ patients and not in DM+CAD− patients. We hypothesized that EPC calcification may be driven by chronic inflammation through stimulation of innate immunity receptors, such as CD14 and TRLs [23, 31, 32], which are expressed by EPCs. Indeed, this pathway has been previously shown to be overactivated in diabetic patients and cardiovascular disease [33]. When EPCs were isolated from DM−CAD− patients and cultured with or without the TRL ligand LPS, expression of bone-related markers was significantly upregulated, also in co-expression with CD34. Of note, OC and BAP overexpression was found also in HUVECs in response to LPS, indicating that this phenotypic change occurs in endothelial cells independently of their origin. This finding should be viewed in light of the postulated cross-talk between endothelial cells and osteoblasts in the regulation of bone turnover [34]. Remarkably, induction of the osteoblast marker RANKL by LPS occurred only in EPCs and not in HUVECs, supporting that EPCs have a stronger tendency toward the osteogenic phenotype. It should be carefully noted that we did not definitely prove that EPC calcified in vitro. While it may be surprising that these cells spontaneously deposit calcium in culture without osteogenic stimuli, the relatively high serum concentration used for EPC isolation (20%) may represent a source of calcium/phosphate. Our data are supported by the recent finding of Liu et al. showing that oxidized low-density lipoprotein and *β*-glycerophosphate induce extensive EPC calcification in vitro [35]. However, further studies are needed to define in greater detail the calcification potential of EPCs in different culture conditions and in vivo.

## 5. Conclusion

Our data have important implications for the interpretation of circulating progenitor cell phenotype in relation to cardiovascular complications of diabetes. Reduced progenitor cell level and impaired endothelial differentiation are currently considered mechanisms whereby diabetes causes endothelial dysfunction and excess vascular damage [11]. Our present data indicating pro-calcific differentiation of circulating progenitors add a new plug to the puzzle and identify a hitherto unrecognized potential mechanism of vascular calcification in diabetes.

## Figures and Tables

**Table 1:** Clinical characteristics of study patients divided according to the presence of DM and CAD. Post hoc analyses: *P < 0.05 in DM+ versus DM−; ^#^ *P* < 0.05 in CAD+ versus CAD−.

Characteristic	DM**−**CAD**−**	DM**−**CAD+	DM+CAD**−**	DM+CAD+	ANOVA *P *
Number	33	19	33	22	—
Age (years)	54.3 ± 3.2	52.4 ± 3.1	61.9 ± 1.8*	67.3 ± 1.4*	<0.001
Sex male (%)	38	79^#^	70*	86	0.001
BMI (kg/m^2^)	24.8 ± 0.4	26.8 ± 1.3	28.3 ± 0.9*	30.6 ± 0.9*	<0.001
SBP (mm Hg)	139.1 ± 4.1	120.4 ± 5.6	143.2 ± 3.2	141.7 ± 4.8	<0.001
DBP (mm Hg)	83.4 ± 2.3	75.3 ± 2.1	85.9 ± 2.1	84.5 ± 2.1	<0.001
Hypertension (%)	30	32	88*	91*	<0.001
Smoking habit (%)	16	26	6	5	0.102
HbA1c (%)	5.2 ± 0.2	5.8 ± 0.1	8.4 ± 0.3*	8.1 ± 0.3*	<0.001
FPG (mg/dL)	87.9 ± 3.9	99.3 ± 4.9	164.3 ± 11.6*	161.4 ± 10.5*	<0.001
T-CH (mg/dL)	203.1 ± 7.1	183.6 ± 11.6	178.9 ± 6.4*	158.5 ± 7.9*	0.002
HDL (mg/dL)	60.0 ± 3.7	49.2 ± 1.9^#^	48.1 ± 2.1*	39.6 ± 2.0^#∗^	<0.001
LDL (mg/dL)	125.2 ± 6.7	105.7 ± 11.3	100.1 ± 5.6*	90.8 ± 6.5	0.008
Triglycerides (mg/dL)	94.7 ± 7.9	144.3 ± 27.9	150.4 ± 17.3*	141.0 ± 12.4*	0.056
Retinopathy (%)	0	0	21*	36*	<0.001
Nephropathy (%)	0	16	6	27^#∗^	0.07
Neuropathy (%)	0	0	21*	27*	0.02
CerVD (%)	21	5	70*	50*	<0.001
PAD (%)	6	0	42*	32*	<0.001
OHA (%)	0	0	76*	68*	<0.001
Insulin (%)	0	0	42*	41*	<0.001
ACEi/ARB (%)	28	95^#^	76*	77	<0.001
Other anti-HT (%)	22	84^#^	55*	77	<0.001
Aspirin (%)	16	79^#^	76*	86	<0.001
Statin (%)	19	68^#^	58*	86	<0.001

BMI: body mass index. SDP, systolic blood pressure. DBP: diastolic blood pressure. FPG: fasting plasma glucose. T-CH: total cholesterol. HDL: high-density lipoprotein cholesterol. LDL: low density lipoprotein cholesterol. CerVD, cerebrovascular disease. PAD: peripheral arterial disease. OHA: oral antihyperglycemic drugs. ACEi: angiotensin conerting enzyme inhibitors. ARB: angiotensin receptor blockers. AntiHT, anti-hypertensive medications.
